# Bioorthogonal Reactions in Bioimaging

**DOI:** 10.1007/s41061-024-00452-1

**Published:** 2024-02-24

**Authors:** Eszter Kozma, Péter Kele

**Affiliations:** Chemical Biology Research Group, Institute of Organic Chemistry, HUN-REN Research Centre for Natural Sciences, Magyar Tudósok Krt. 2, Budapest, 1117 Hungary

**Keywords:** Bioorthogonal, Fluorescent labeling, Fluorogenic probes, Live cell imaging

## Abstract

Visualization of biomolecules in their native environment or imaging-aided understanding of more complex biomolecular processes are one of the focus areas of chemical biology research, which requires selective, often site-specific labeling of targets. This challenging task is effectively addressed by bioorthogonal chemistry tools in combination with advanced synthetic biology methods. Today, the smart combination of the elements of the bioorthogonal toolbox allows selective installation of multiple markers to selected targets, enabling multicolor or multimodal imaging of biomolecules. Furthermore, recent developments in bioorthogonally applicable probe design that meet the growing demands of superresolution microscopy enable more complex questions to be addressed. These novel, advanced probes enable highly sensitive, low-background, single- or multiphoton imaging of biological species and events in live organisms at resolutions comparable to the size of the biomolecule of interest. Herein, the latest developments in bioorthogonal fluorescent probe design and labeling schemes will be discussed in the context of in cellulo/in vivo (multicolor and/or superresolved) imaging schemes. The second part focuses on the importance of genetically engineered minimal bioorthogonal tags, with a particular interest in site-specific protein tagging applications to answer biological questions.

## Introduction

Efforts in the past decades have resulted in substantial hardware developments in fluorescence microscopy, which have revolutionized the field of optical imaging [1]. The emerged deterministic and stochastic superresolution microscopy (SRM) methods allow the visualization of cellular structures in their native context at resolutions never seen before. Optical imaging-aided interrogation of live systems facilitates better understanding of cellular processes. Through unraveling new genetic or metabolic pathways, or drug mechanism of action, live-cell imaging schemes can have considerable implications in drug discovery, as well. To take full advantage of the latest hardware developments and to address more complex questions, the growing demands of superresolution microscopy (SRM) methods need to be addressed [2]. These needs have defined recent research trends in the field of fluorescence probes, as it is rather the lack of suitable markers that can be considered as the major limitation in fluorescence imaging. Even in case of probes with ideal photophysical characteristics, such as high molar extinction coefficient, fluorescence quantum yield, and photostability, the signal-to-noise ratio achievable is often impaired by the fluorescence of endogenous fluorophores, e.g., reduced nicotinamide adenine dinucleotide (NADH), flavins, porphyrins, aromatic amino acids, etc. [3]. These autofluorescence-related issues can be efficiently addressed by selecting dyes with excitation bands in the red, far-red, or near-infrared range. Alternatively, scaffolds with large Stokes shifts can also be applied, as the excitation and emission maxima of natural fluorophores are very close to each other. Synthetic probes can also display considerable background fluorescence, which may compromise the sensitivity and resolution. This problem may be reduced by applying several washing cycles to remove nonspecifically adsorbed probes from the samples. However, this time-consuming process considerably delays the acquisition of microscopic data and prevents the rapid detection of proteins of interest (POIs) immediately after labeling reactions [4]. This is crucial, e.g., in case of proteins with rapid turnover rates. Fluorogenic markers that exist in a quenched state until being transformed to an emissive form in response to a specific trigger efficiently minimize background fluorescence while keeping the number of washing cycles low [5].

A further fundamental challenge for probes is posed by the need for selective and site-specific modification of target structures while keeping the size of the probe as small as possible to minimize perturbation and reduce linkage errors, i.e., displacement of the probe from the tagging site of the target protein [2, 6]. Addressing the challenge of selective and site-specific labeling of biomolecules, bioorthogonal reactions have become indispensable tools to install small organic, minimally perturbing markers onto various cellular targets. The pioneering work of Bertozzi that applied phosphines (i.e., in a Staudinger–Bertozzi ligation) for bioorthogonal tagging of surface glycans of cells led to the postulation of bioorthogonality in 2002 [7]. Since then, covalent modification of biomolecules with various probes and tags has become one of the hallmarks of bioorthogonal chemistry applications. A recent statistical analysis of publication data between 2010 and 2020 revealed that papers demonstrating the robustness of bioorthogonal chemistry-aided labeling and imaging of biomolecules in live organisms contribute with the highest percentage to reports in the field of bioorthogonal chemistry applications [8]. Bioorthogonal modification schemes allow for the modification of virtually any biomolecules. Indeed, there are examples of the selective labeling of various biopolymers (e.g., carbohydrates [9, 10], nucleic acids [11, 12], lipids [13, 14], and proteins [15, 16]) or small-molecular ligands [17, 18]. Yet, protein-related bioorthogonal labeling schemes are, by far, the most popular, possibly owing to the advanced technology for the incorporation of bioorthogonal handles into proteins [8]. Further trends related to the bioorthogonal reactions applied for covalent installation of markers can also be identified: not long after its introduction, the Staudinger ligation was overwhelmed by copper-catalyzed azide–alkyne cycloaddition (CuAAC) [19, 20] or rather by its more biocompatible strain-promoted version (SPAAC) [21, 22]; however, these reached a plateau by 2016. Meanwhile, several bioorthogonal ligation reactions were reported [8, 23–25], yet it is the inverse electron-demand Diels–Alder reaction (iEDDA) of tetrazines and strained alkenes/alkynes [26, 27] that is still on the rise in terms of the bioorthogonal ligation step applied for the installation of markers onto biomolecules [8]. It should be noted, however, that the easy metabolic incorporation of azide-appended chemical reporters keeps SPAAC-based tagging schemes in the race.

In general, bioorthogonal tagging reactions follow a two-step scheme in which the biomolecule of interest is first modified with a so-called chemical reporter bearing a bioorthogonal function by means of metabolic incorporation, protein engineering, or enzymatic action [23, 25, 28, 29]. Such site-specifically bioorthogonalized systems are then targeted selectively with a probe (marker, affinity label, etc.) harboring a complementary bioorthogonal function. Such modulated biomolecules are subsequently used to interrogate biological systems to provide deeper insight, e.g., into biomolecular processes. While markers with various modalities are applied in bioorthogonal tagging schemes, the present discussion is limited to fluorescent markers. Examples for imaging by further modalities, e.g., PET (SPECT), are covered in Chapter 8.

Since the introduction of highly advanced superresolution methods such as MINFLUX [30] and MINSTED [31], the means of the labeling method became another major limiting factor on resolution. The past decades have brought about substantial developments in the field of labeling techniques. Indeed, the fusion of engineered proteins such as fluorescent proteins (FPs) or self-labeling tags is routinely accomplished. However, the relatively large size (2–5 nm) of these tags often impairs the resolution through linkage error [2]. Reducing the linkage error became a major issue in superresolution imaging techniques [32–35]. Nanobodies with dimensions as small as 1.5 × 2.5 nm^2^ may offer alternatives for targeting, but the palette of their targets is rather small [36]. Bioorthogonal labeling schemes that rely on the incorporation of minimal tag bioorthogonalized building blocks and suitably functionalized probes allow minimal linkage error, and very importantly, these are not limited to proteins [37–39]. Synthetically tailored organic fluorophores offer much smaller size and allow greater flexibility in terms of the site specificity of labeling, often providing improved photophysical properties such as a wider spectral range and greater photostability or brightness compared with, e.g., FPs [2, 33]. Furthermore, tailored synthetic probes may also address challenges such as auto- and background fluorescence [2]. As stated above, fluorogenic probes reduce the background signal of nonspecifically adsorbed dyes while avoiding the need for extensive washing cycles [2, 40–42]. In this latter regard, fluorogenic probes that are switched on in response to a bioorthogonal reaction are of major interest [41]. In multicolor labeling schemes, further problems are encountered owing to chromatic aberration, which can be minimized with sophisticated image processing [43, 44] or by applying probes with considerably different Stokes shifts [45–47].

In the last decade, research trends in bioorthogonal reaction-aided fluorescent labeling schemes have focused on the development of bioorthogonally controlled fluorogenic probes. Herein, we venture to provide the reader with a comprehensive overview of these latest developments. First, we discuss recent progress in the field of bioorthogonal fluorogenic probe design, while the second part lists the highlights of genetically incorporated minimal bioorthogonal tags with a special focus on site-specific protein labeling applications to answer biological questions.

## Fluorogenic Probes: General Considerations

Fluorogenic probes have the salient feature of existing in a weakly (ideally non) emissive form at a given wavelength until they are bound in a specific ligation step to their target. Fluorescent cores can be rendered fluorogenic by various means. Indeed, there are probes described in literature that are modulated by photoinduced electron transfer, energy transfer, electronic changes as a result of enzymatic action, alterations in environment polarity, or structural changes [48]. Herein, we restrict our discussion to probes that are modulated by bioorthogonal motifs. The extent of fluorogenicity can be characterized, e.g., by the ratio of the fluorescence intensity, quantum yield, or brightness of the conjugated and free forms at a given wavelength. Comparison of brightness is probably the most practical from the imaging point of view, as it also encompasses changes in absorptivity upon conjugation. Generally, the larger the increase in the respective term, the higher the achievable contrast and consequently the less it is required to wash off unreacted probes. Experience suggests that even one order of magnitude difference is sufficient to differentiate specifically bound from freely diffusing or non-specifically adsorbed species [49, 50].

**Fig. 1:**
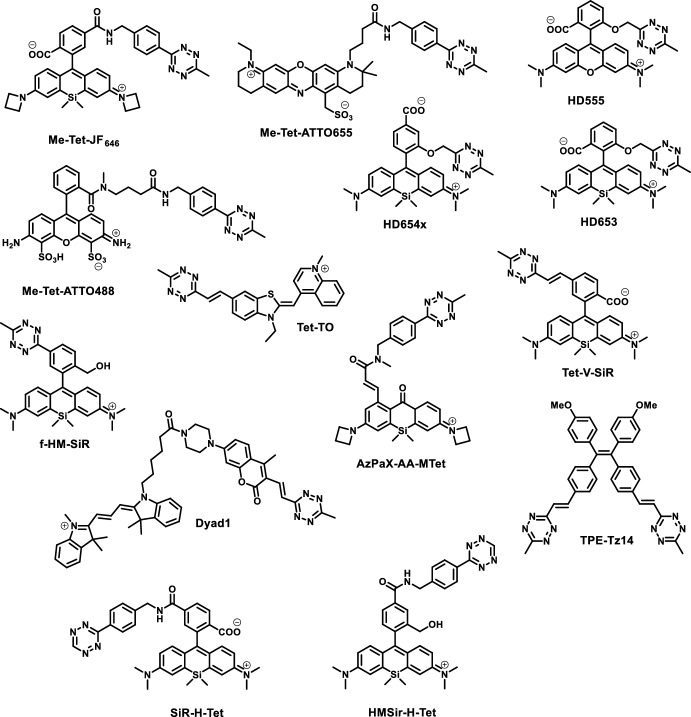
Tetrazine-appended fluorogenic probes discussed herein

There are several bioorthogonal functions that can render probes fluorogenic. These examples include tetrazoles [51, 52], cyclooctynes [53, 54], linear alkynes [55], sydnones [56], pyrones [57], and most notably, azides [10, 58] and tetrazines [59, 60]. Recent examples, however, suggest that the tetrazine motif is the most widely applied in the design of bioorthogonally applicable, fluorogenic probes owing to its fast reaction kinetics and good quenching potential. This two-in-one combination of being a bioorthogonal handle and a quencher of fluorescence was exploited in the design of various fluorogenic scaffolds involving coumarin [59], boron-dipyrromethene (BODIPY) [60], phenoxazine [61], rhodamine [49], and cyanine-based [62], bioorthogonally applicable fluorogenic probes. Most tetrazine-appended probes apply either a 3-methyl-6-phenyl-1,2,4,5-tetrazine (Me-Tet) or a 3-phenyl-1,2,4,5-tetrazine (H-Tet) as the quencher moiety. The major difference between the two tetrazines is their different reactivity and chemical stability. While H-Tet reacts approximately 30 times faster in click reactions, it exhibits lower chemical stability under physiological conditions [63]. There are several means for the efficient installation of the tetrazine moiety onto fluorescent scaffolds to render them fluorogenic. In this respect, Heck [64] and Suzuki [62] cross-coupling reactions readily decorate fluorophores with tetrazine through vinylene and phenylene linkage, respectively. Stille coupling reactions, on the other hand, are demonstrated to directly connect tetrazine frames to aromatic scaffolds [49] (Fig. 1).

### Ways to Improve the Fluorogenicity of Tetrazine-Modulated Probes

Tetrazines exert their quenching effects through various mechanisms [65]. Generally, the emission intensity of ultraviolet (UV)/blue/green excitable cores, e.g., coumarins and BODIPYs, is very effectively modulated by energy (e.g., Förster resonance energy transfer (FRET) [13, 66] or through-bond energy transfer (TBET) [59, 60]) or electron transfer (PET [49]) processes. Direct conjugation of tetrazines to a UV/blue/green-excitable fluorescent scaffold may also result in excellent quenching via internal conversion (IC) to a dark lowest-lying excited state [67–69]. A recent report shows that IC-based quenching can account for quenching in case of orange-emitting Cy3 cores connected to Me-Tet directly or via vinyl linkage, although with much less efficiency [70]. These examples highlight the strengths but also the limitations of tetrazine-based modulation strategies in terms of ligation schemes and fluorogenic behavior, as the quenching efficiency of the tetrazine declines dramatically toward the biologically preferred, red range of the spectrum [71]. This prompted research directions that aimed at improving the fluorogenicity of tetrazine-modulated red-excitable probes. Initial attempts involved the installation of two tetrazines onto orange-excitable Cy3 frames. Conjugation experiments with doubly cyclooctynylated designer peptide sequences fused to target proteins revealed somewhat improved fluorogenicity values, possibly due to a superimposed fluorescence increase originating from the formation of a conformationally constrained cyclic product. The moderate increase and the need for the rather complicated incorporation of a bis-cyclooctynylated peptide fusion tag into proteins, however, marginalized these approaches [72].

Further attempts for multiply fluorogenic systems combined tetrazine-based reaction-driven fluorogenicity with other fluorogenic mechanisms. These resulted in vinyltetrazine-appended 2′-carboxy silicorhodamines that featured an additional, polarity-driven valence tautomerism between a fluorescent zwitterionic and a dark spirocyclic form. In this pioneering example, the combined quenching mechanisms gave rise to substantially improved fluorescence enhancement (FE) values (i.e., 22 fold) [50] in comparison with congeners possessing either one of the quenching effects [49, 73] separately. Superresolved (GSDIM) imaging of vimentin^116TAG→BCN^mOrange-expressing live COS7 cells labeled with Tet-V-SiR allowed an improved, 28 nm resolution compared with the reporter channel, although its use was not demonstrated in live cells.

**Fig. 2:**
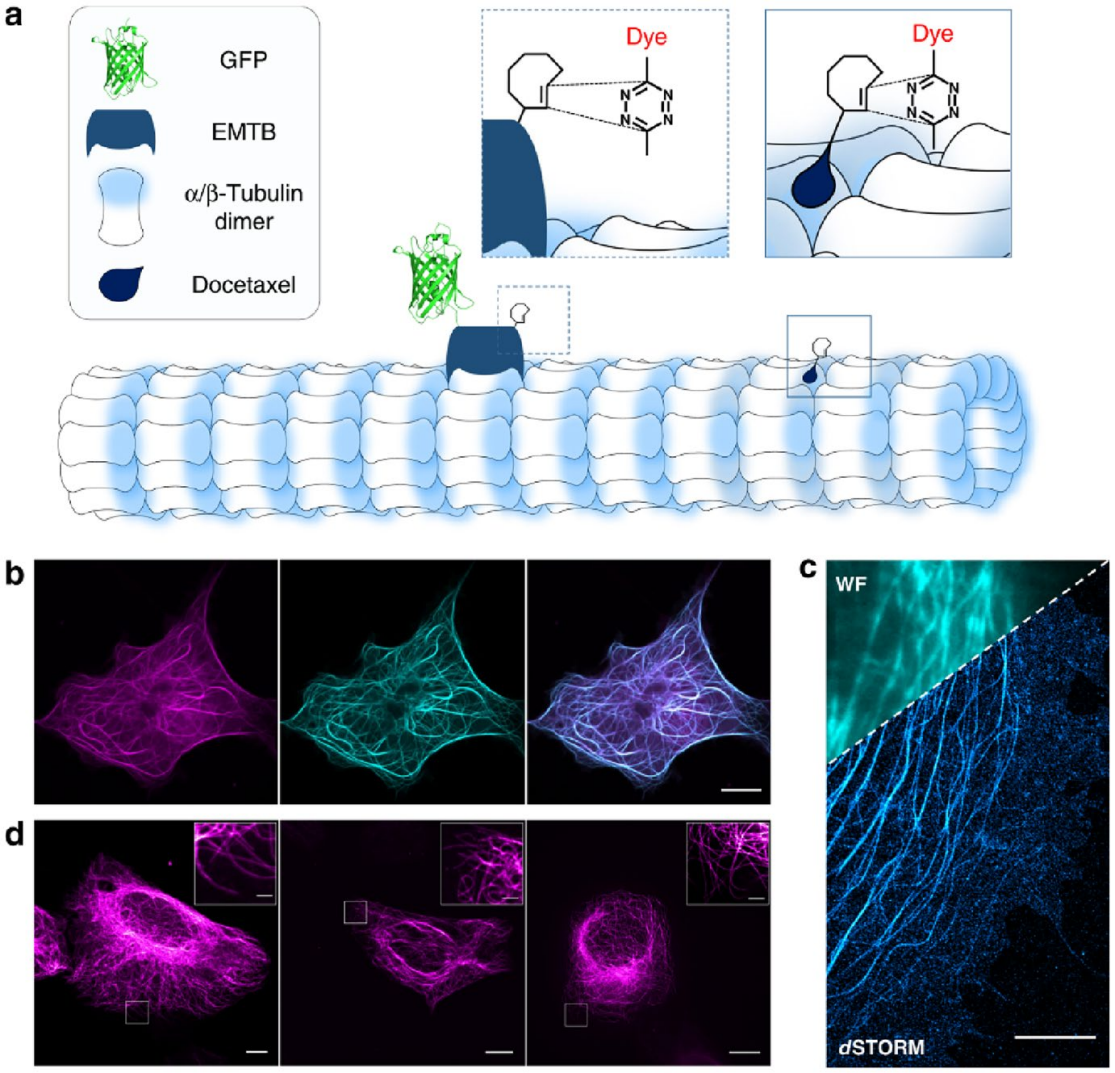
Intracellular live-cell labeling and imaging of clickable microtubule-binding domain of ensconsin (EMTB) in COS-7 cells. **a** Scheme of incorporation of unnatural amino acid TCO*-Lys into the microtubule-associated protein EMTB^K87TAG^−3xGFP via GCE and bioorthogonal labeling with a tetrazine dye. Alternatively, microtubules can be labeled in living cells with docetaxel-TCO followed by click labeling with a cell-permeable tetrazine dye. **b** Live-cell confocal fluorescence images of the construct EMTB.^K87TAG^−3xGFP labeled with 3 µM of the membrane-permeable SiR-H-Tet for 10 min (GFP, cyan; SiR, magenta; and overlay). **c** Single-molecule localization microscopy image of the same construct. Cells were fixed and labeled with 3 µM HMSiR-H-Tet and then imaged in PBS (pH 7.4). The upper left corner shows an overlay with the corresponding wide-field (WF) image. **d** Live-cell confocal, rescan confocal, and SIM fluorescence images (from left to right) of U2OS cells treated with 10 µM docetaxel-TCO for 30 min and labeled with 10 µM SiR-H-Tet for 10 min. The inserts in the upper right corner show expanded views of the marked regions, demonstrating the improved spatial resolution. Scale bars, 5 µm (**b**–**d**), 1 µm (expanded views). (Adapted from Ref. [74] with permission. Copyright © 2019, The Authors, Published by Springer Nature)

Sauer and coworkers have studied a fine set of fluorogenic tetrazine appended oxazine and rhodamine dyes (e.g., Me-Tet-JF_646_, Me-Tet-ATTO488, and Me-Tet-ATTO655) [74]. Their comprehensive and instructive study compared these tetrazinylated probes in terms of fluorogenic behavior but also elaborated the obtainable resolution of different labeling methods. More particularly, they imaged the very same proteins either by signals of a fused FP or small-molecule tetrazine probes anchored to the protein through a minimal tag (i.e., TCO*-Lys) (Fig. 2). Besides highlighting the importance of linkage error, a further valuable conclusion was to reveal a stacking interaction-aided electron transfer between the oxazine core and a flexibly linked tetrazine, which may be accounted for an unexpectedly effective fluorogenic behavior. The Wombacher group followed this lead and utilized this knowledge in their design concept to push the limits of fluorogenic tetrazine–xanthene probes to previously unseen FE values in the given spectral range [75]. In their report, they enclosed a set of red and far-red fluorogenic tetrazine probes dubbed HDyes (HD555, HD653, and HD654x), where a tetrazine unit was flexibly linked to the xanthene core at minimal distance, allowing efficient fluorescence quenching via a Dexter electron exchange mechanism. One of their (silico)rhodamines, HD555, reached a fluorescence increase of up to 123-fold compared with the IEDDA reaction.

**Fig. 3:**
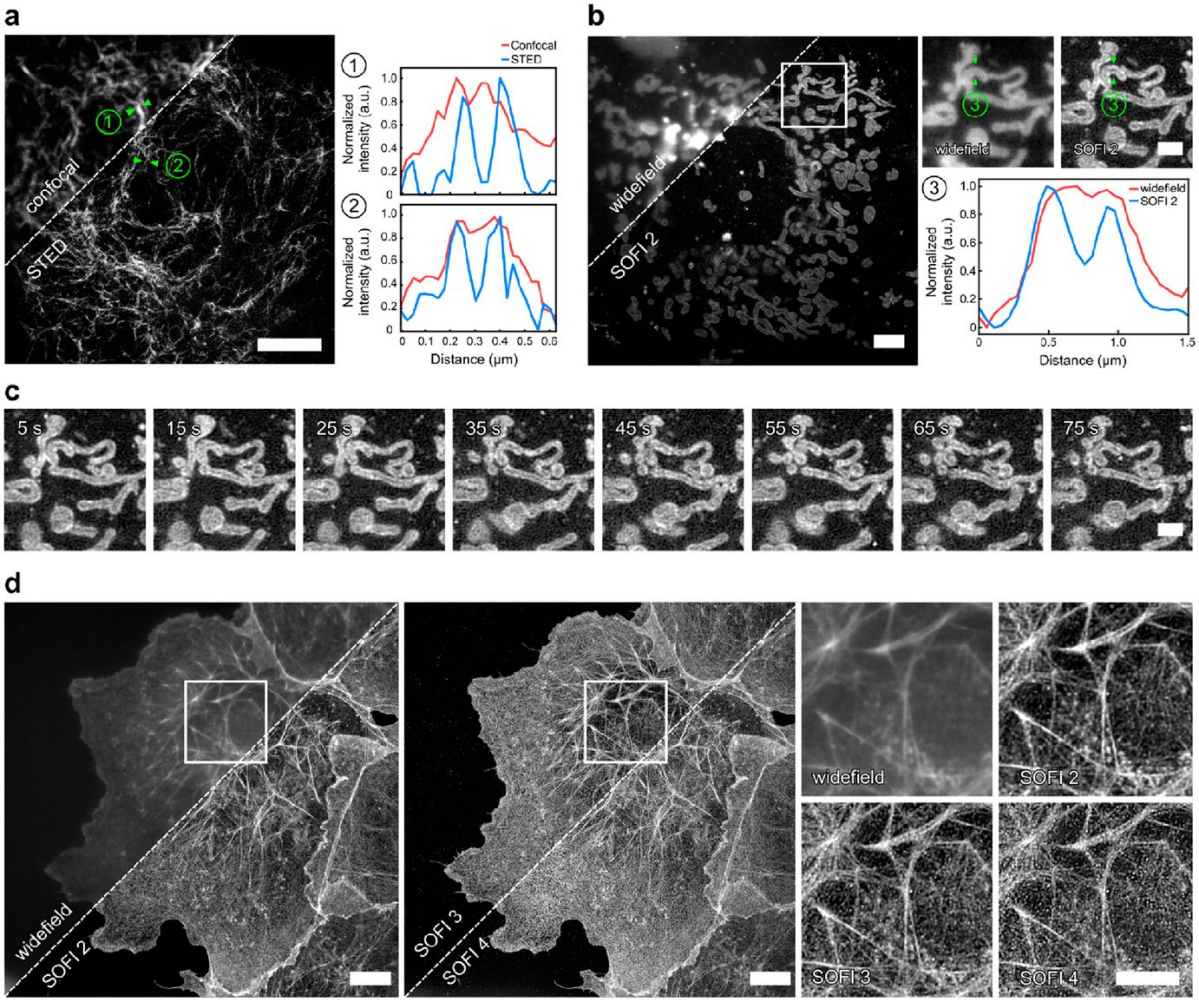
Superresolution microscopy with HDyes. **a** Live-cell confocal and STED microscopy of COS-7 cells expressing pVimentin^N116TAG→BCN^ labeled with HD653 with intensity line profiles showing the resolution improvement. Scale bar, 5 μm. **b** Live-cell wide-field (temporal average of series) and second-order SOFI of mitochondria in COS-7 cells transiently expressing TOMM20-mCherry–HaloTag labeled with sb-HD656 via Halo–BCN. Scale bar, 5 μm. The close-ups correspond to the ROI indicated in the overview image. The cross-sectional profile of the averaged wide-field image and second-order SOFI shows the improved image contrast and resolution. Scale bar, 2 μm. **c** Time course of second-order SOFI corresponding to the ROI indicated in (**b**), showing the movement of mitochondria with a time resolution of 10 s. Image acquisition: 500 frames per time point, 20 ms exposure time, 635 nm laser, 140 W cm^−2^. The images are representative of more than five cells from two independent experiments. **d** High-order SOFI imaging of sb-HD656-labeled f-actin in fixed COS-7 cells, showing SOFI analysis up to fourth cumulant order. Image acquisition: 20,000 frames, 50 ms exposure time, 635 nm laser, 275 W cm^−2^. The images are representative of six cells from two independent experiments. Scale bars, 10 μm. (Adapted from Ref. [75] with permission. Copyright © 2021 The Authors. Published by American Chemical Society)

The membrane permeability of their probes was also fined-tuned via *o*- or *p*-positioning of a pendant carboxyl group. A number of live-cell applications were demonstrated in combination with noncanonical amino acid (BCN-Lys) incorporation or Halo tag fusion (Halo-BCN) as means for dienophile tagging of proteins. Such bioorthogonal fluorogenic labeling schemes allowed extra/intracellular multicolor, no-wash labeling of target structures with subsequent live-cell STED as well as superresolution optical fluctuation imaging (SOFI) (Fig. 3) [75]. Liu and Wu followed a similar design strategy and installed an aminotetrazine at the *meso* position of various far-red, NIR-emitting xanthenium and cyanine cores [76]. The authors concluded that the tetrazine exerts its quenching effect via PET to reach remarkable, up to 1400-fold fluorescence turn-on ratios in the 586–806 nm emission range.

Like 2′-carboxyrhodamines, 2′-hydroxymethyl or 2′-hydroxyethyl rhodamines are also prone to keep an equilibrium between a colorless, nonfluorescent spirocyclic and a fluorescent open form [77]. The equilibrium is highly dependent on the pH of the medium and is characterized by a term called p*K*_cycl_, which corresponds to the pH at which the absorbance is half of the maximum value. The p*K*_cycl_ can be synthetically fine-tuned to suit the purposes of the actual experiment. For example, probes that possess p*K*_cycl_ below ca. 5.5 show very low intensity fluorescence at physiological pH, as the nonfluorescent form is dominant in this pH range. The equilibrium process allows stochastic formation of a small population of the emissive form, giving rise to spontaneous blinking without the need for additives such as blinking buffers and thus enabling stochastic methods in vivo [78]. The interplay between the dark and emissive forms was used in bioorthogonal labeling schemes as well. For example, Schepartz and Toomre took advantage of the shifted ON/OFF ratio of a tetrazine-decorated 2’-hydroxymethylrhodamine probe developed by Johnsson et al. [73], by anchoring it in a hydrophobic environment [79]. By selectively tagging membranes using bioorthogonal tetrazine chemistry, the authors were able to conduct three-dimensional (3D) imaging of endoplasmic reticulum dynamics in a live cell using SMLM. Their results demonstrated how long-time-lapse superresolution imaging could be achieved with increased labeling density and reduced emission intensity in a hydrophobic environment. Later, the authors took further advantage of the extended time imaging enabled by these probes in two-color imaging of organelles [80].

**Fig. 4:**
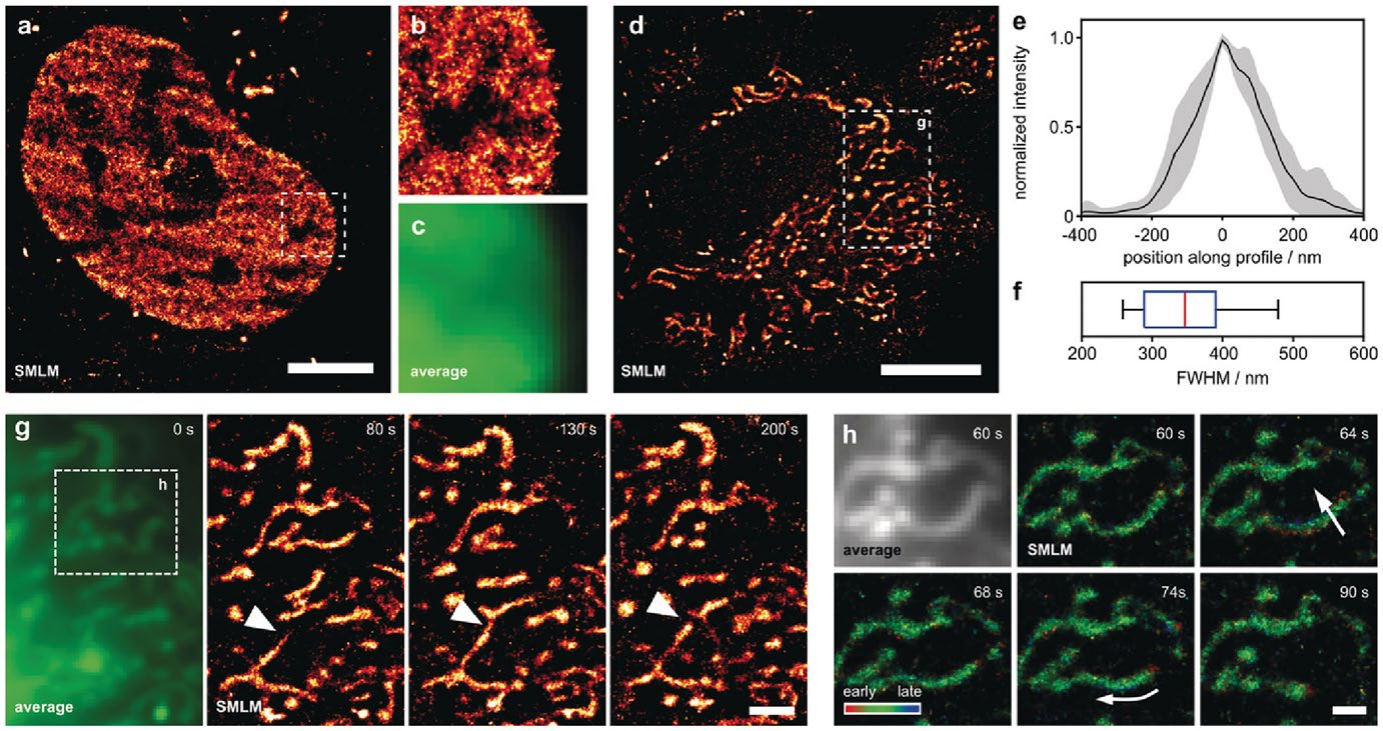
f-HM-SiR reveals cellular dynamics in live HeLa cells with improved resolution. **a** HeLa cells transiently expressing H2A-HaloTag were incubated with HTL-BCN (10 μm), washed, and labeled with f-HM-SiR (2 μm). A reconstruction from 333 frames corresponding to 6.67 s acquisition time is shown. **b** Zoom-in of boxed region in (**a**). **c** Corresponding averaged image for boxed region in (**a**). **d** HeLa cells were incubated with TPP-BCN (10 μm), washed, and labeled with f-HM-SiR (2 μm). Reconstruction from 500 frames (10 s) is shown. **e** Averaged cross-sectional profiles from mitochondrial tubules after alignment, ± 1 standard deviation, *n* = 11. **f** Width of individual profiles shown in (**e**). **g** Zoom-in of boxed region in (**d**). Average (left) and reconstructions from 500 frames at different time points (right). Arrowhead indicates mitochondrial fusion event. **h** Average (top left) and reconstructions of boxed region in (**g**). Localizations are colored with respect to their relative time of appearance within a single reconstruction. Scale bars: **a** 5 μm, **d** 10 μm, **g** 2 μm, **h** 1 μm. (Adapted from Ref. [82] with permission. Copyright © 2019 The Authors. Published by Wiley–VCH Verlag GmbH & Co. KGaA)

While in the previous example the bioorthogonal ligation step only directed the probe to a suitable environment, there are instances where the tetrazine does more than just positioning. Subtle electronic changes as a result of transformation of substituents at the xanthenium unit by enzymatic action also effectively change the emission intensity owing to shifted equilibrium as a result of altered p*K*_cycl_ values [81]. Such modulation of the equilibrium process was achieved in response to an iEDDA reaction by the Wombacher group [82]. Their hydroxymethyl-appended far-red-emitting silicorhodamine was decorated with a tetrazine unit at the upper ring of the probe (f-HM-SiR). The strongly electron-withdrawing character of the appended tetrazine facilitated deprotonation of the hydroxymethyl arm, promoting subsequent spirocyclization. Transformation of the tetrazine to the respective iEDDA product changed the equilibrium process as a result of the decreased electron-withdrawing character of the (dihydro)pyridazine product. They have demonstrated that the tetrazine form of their probe featured an equilibrium constant of p*K*_cycl_ 4.0, indicating that the dark spiroether form prevails (99.9%) at physiological conditions. The iEDDA product, on the other hand, showed a significant shift to p*K*_cycl_ 5.2, allowing an increase of the proportion of the fluorescent form but still remaining below 1% at physiological pH (99.4% spiroether), which is necessary for stochastic blinking [82]. The authors demonstrated the use of such fluorogenic, spontaneously blinking probes in no-wash, live-cell labeling SMLM imaging schemes (Fig. 4).

**Fig. 5:**
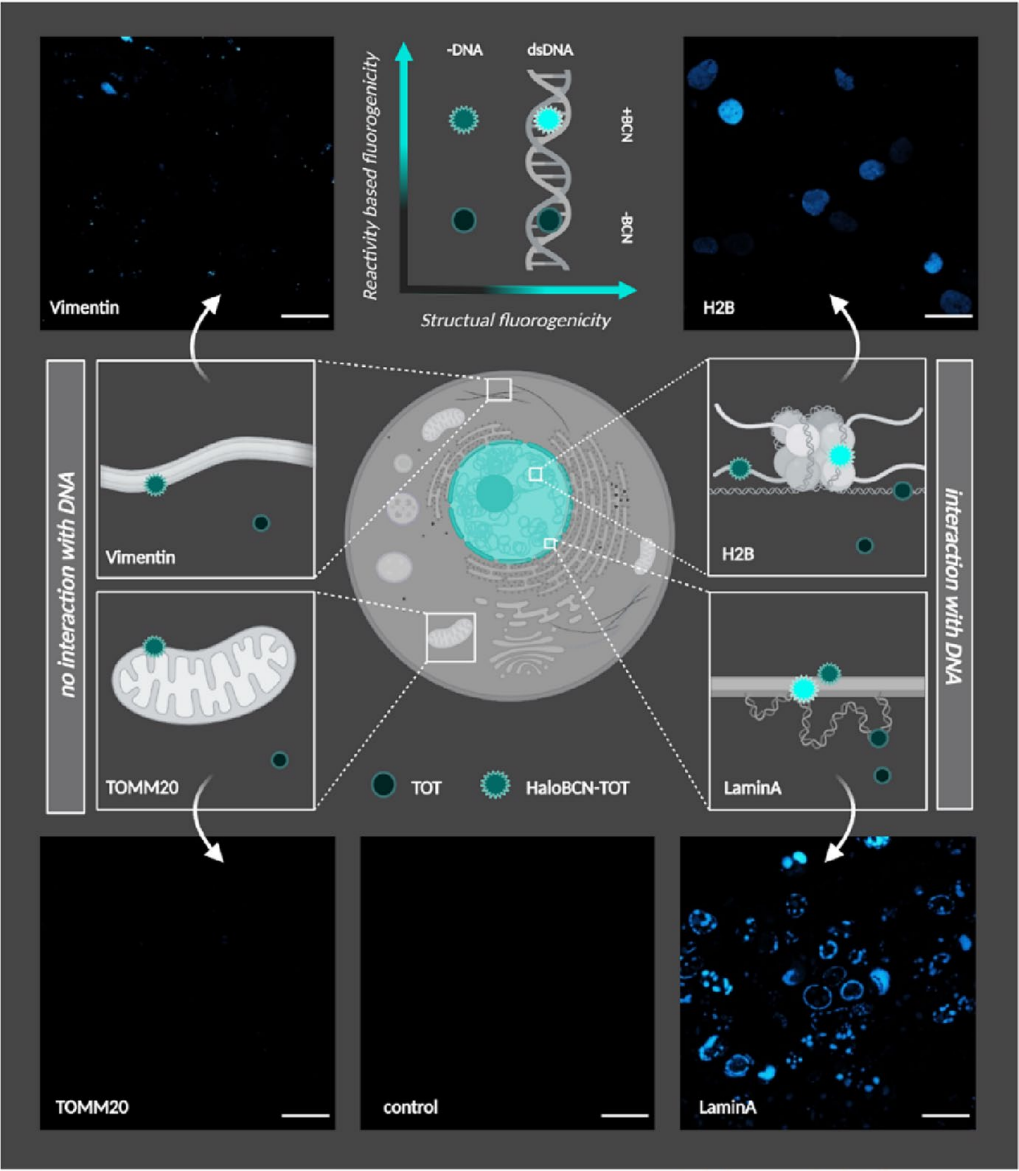
Confocal microscopy images of transfected HEK293T cells expressing the DNA interacting nuclear proteins H2B-HaloTag (top, right) or LaminA-HaloTag (bottom, right) and the non-DNA interacting cytosolic proteins vimentin-HaloTag (top, left) and TOMM20-HaloTag (bottom, left) after treatment with Halo-BCN (3 µM) and Tet-TO (6 µM). The color calibration code shows the fluorescence intensity levels of Tet-TO (top, middle). The control image refers to Tet-TO-treated non-transfected cells (bottom, middle). Spectral detection: *λ*_exc_: 488 nm/*λ*_em_: 500–800 nm; scale bar, 25 µm. Created with BioRender.com. (Adapted from Ref. [83] with permission. Copyright © 2022 The authors. Licensee MDPI, Basel, Switzerland)

Rhodamine cores are not the only ones prone to show structurally driven, environment-sensitive fluorogenic behavior. DNA intercalator probes, such as thiazole orange (TO) and congeners, are known to be nonfluorescent in aqueous medium in their free form owing to a fast rotation-driven deplanarization at a methine bridge holding together the quinoline and benzothiazole units. Our group decorated the TO core with a tetrazine handle (Tet-TO) to render it double fluorogenic. It was expected that the two mechanisms, i.e., tetrazine-based quenching and structural quenching owing to fast rotation, act independently and either one can lead to quenched fluorescence. This implies that both the iEDDA reaction and DNA intercalation are required for complete turn-on, suggesting an AND type of logic gate. To take advantage of these features in the detection of protein–DNA interactions, HaloTag fusion constructs of DNA binding proteins (i.e., HB2 and LaminA) and cytosolic proteins (TOMM20 and vimentin) were tagged with a cyclooctyne-derivatized HaloTag substrate (Halo-BCN) and then treated with probe Tet-TO. The characteristic structure of the BCN-tagged proteins appeared via fluorescence detection only when the probe was anchored to nuclear proteins via iEDDA. The presence of only one of the criteria, as in the case of cytosolic proteins, was not enough to arm fluorescence (Fig. 5) [83].

Further attempts to improve fluorogenicity toward the red range include the concept of relayed fluorogenicity. This design allowed the transformation of the excellent fluorogenicity of coumarins to flexibly attached orange/red-emitting cyanine frames via Förster resonance energy transfer (FRET)[84]. Such a FRET-based relay mechanism resulted in improved yellow/red fluorogenicities of cyanines, allowing improved signal-to-noise in live-cell labeling and imaging schemes. Furthermore, the fluorogenic dyads possessed increased photostabilities, enabling longer STED exposure. Moreover, the large apparent Stokes shifts of the resulting fluorogenic dyads in combination with the parent fluorogenic coumarin allowed three-color imaging using a single excitation source, minimizing excitation-based chromatic aberration.

Tetrazines are able to modulate photoresponsivity in a broader sense. Besides fluorescence, ^1^O_2_ sensing or photodissociation abilities can also be quenched by an appended tetrazine unit as were demonstrated by iEDDA activatable PDT agents [85] or photocages [68]. This feature was harvested by Hell, Bossi and collegues when they applied the tetrazine motif as a caging unit of photoswitchability. They combined a photoactivatable xanthone (PaX) core with a tetrazine unit to access a small-sized probe for the selective labeling of unnatural amino acids (BCN-Lys) introduced by genetic code expansion to proteins. The minimal bioorthogonal tag and the small probe allowed to take full advantage of the nanometer precision of MINFLUX and MINSTED [86]. Sequential switching of probes between an ON and an OFF state is a popular super-resolution method to distinguish adjacent fluorophores at molecular-scale proximities. Transition between the ON and OFF states is commonly achieved with by means of complex imaging buffers that are incompatible with live-cell imaging [87]. Live cell stochastic methods require the use of spontaneously blinking fluorophores, such as the above mentioned HMSiR, photoswitchable dyes, or photoactivatable dyes [88, 89]. In their paper, the rate of photoactivation of the PaX core was efficiently attenuated by the appending tetrazine group by quenching the xanthone’s excited state via electron transfer and thus precluding intersystem crossing and subsequent fluorophore formation. They linked several PaX cores with H-Tet and Me-Tet units via various linkers to screen for the best constellation of the key units in terms of difference between photoactivation rates and quantum yields between the tetrazine and the respective IEDDA products with BCN. They identified AzPaX-AA-MTet, which furnished ideal combination of features required for nanoscopy.

**Fig. 6:**
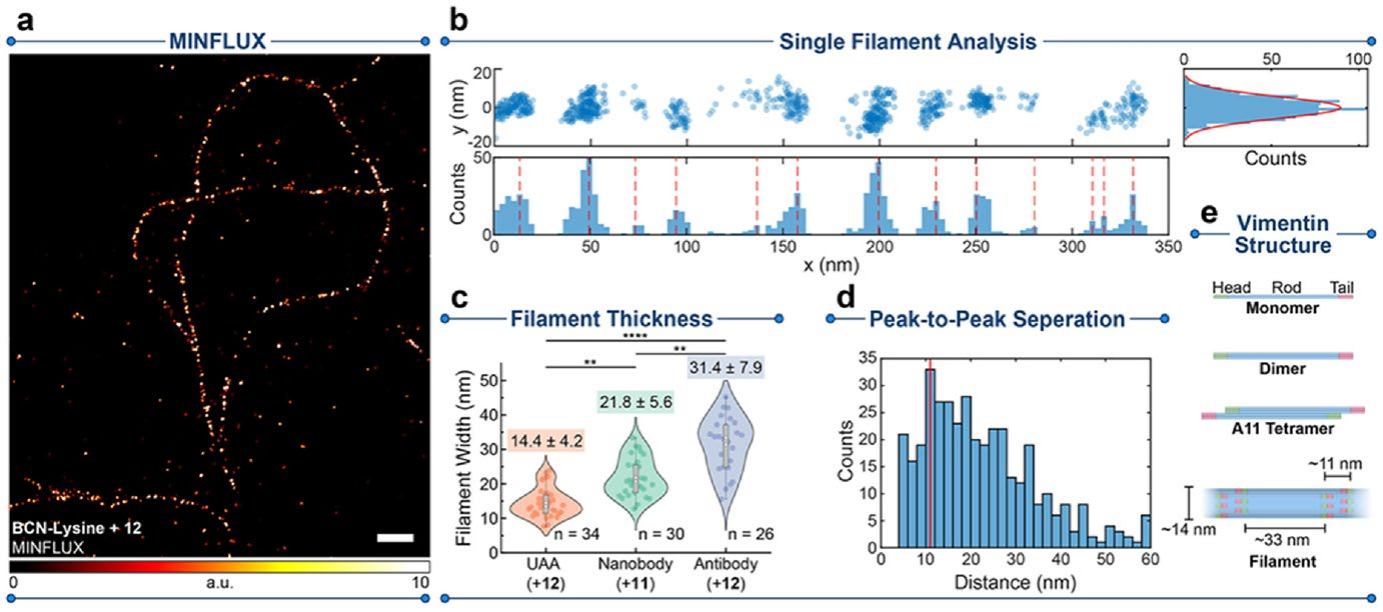
MINFLUX characterization of linkage-error-free labeling. **a** MINFLUX image of vimentin filaments in COS-7 cells incorporating endo BCN-L-lysine and labeled with compound AzPaX-AA-MTet. **b** Single filament analysis of the linearized filament marked in panel a; distributions of localizations along the filament length (*x*) and width (*y*) were used to determine peak-to-peak separation of localization domains and the fwhm of the filament segment, respectively. **c** Distribution of filament thicknesses of vimentin segments labeled with different strategies, including UAAs + AzPaX-AA-MTet, anti-GFP nanobodies + compound MePaX-AA-MTet, and indirect immunofluorescence + AzPaX-AA-MTet in transfected COS-7 cells (as described above). The distribution mean ± SDs are given above the violin plots, and n designates the number of analyzed filaments segments. **P < 0.01; ****P < 0.0001 using the Kruskal–Wallis and the Dunn method for posthoc correction for multiple comparisons. **d** Peak-to-peak separations of localization domains in vimentin filaments labeled with UAAs. **e** Structure of the vimentin according to recent literature the obtained values shown. Scale bar: 200 nm. (Adapted from Ref. [86] with permission. Copyright © 2023 The Authors. Published by American Chemical Society)

The compromise hit molecule contained an azetidine-bearing PaX (AzPax) scaffold for high quantum yield of the final photoactivated IEDDA product, a secondary acrylamide (AA) linker for minimal size and to reach slow photoactivation rate, both before and after reaction with BCN (for greater activation control during imaging) and a Me-Tet (MTet) for improved stability. They then demonstrated the power of their linkage error free labeling in MINFLUX nanoscopy in COS-7 cells transiently expressing a vimentin-mCerulean3 fusion construct carrying a N116TAG mutation for BCN-Lys incorporation. Following treatment of the cells with AzPaX-AA-MTet and subsequent activation with 405 nm illumination, the individual fluorophores were localized with a median precision of 2–3 nm. The average full-width half-maximum (fwhm) of linearized filament segments suggested an average value of 14 nm, which is in excellent agreement with cryogenic electron microscopy/electron tomography data (Fig. 6). Distribution of filament thicknesses of vimentin segments labeled by different means i.e., genetic incorporation of BCN-Lys, nanobody tagging or immunofluorescence highlighted the importance of minimal tag incorporation strategies in super-resolution imaging (Fig. 6c).

Research of aggregation induced emission (AIE) governed luminogens (AIEgens) represents an emerging field [90]. The most typical AIEgen, tetraphenylene (TPE) exhibits virtually no fluorescence in solution in its free form, due to fast rotation of the substituents. Restricting these rotary motions by means of anchoring [91] or aggregation, however, leads to the appearance of intense emission. Such AIEgens have found widespread applications including biosensors, optoelectronic devices, or functional materials and fluorogenic probes. Not surprisingly, bioorthogonal chemistry also had implications to AIEgens. Kim and Kim discovered that emission of a set of aggregated Kaleidolizines (KIz) is efficiently quenched by a directly connected tetrazine unit through internal conversion to a dark state. This rendered the studied KlzTets non-fluorescent in their free or aggregate forms until the tetrazine unit is intact. When, however, it was transformed to the respective dihydropyridazine in a iEDDA scheme with TCO, the aggregates became fluorescent. This suggested the presence of reactivity-based and structural fluorogenicity similarly to the previous examples e.g., to Tet-TO. Co-administration of mitochondria targeting triphenylphosphine derivatives bearing a TCO and KlzTet resulted in highly specific mitochondria staining [92].

The very same concept was applied to TPE as well by Tian and co-workers [93]. The fact that the studied Tet-TPE probes did not fluoresce in their aggregated form efficiently addressed a problem present in AIEgen imaging, i.e., generation of false positive signal due to non-specific aggregation. They further increased fluorogenicity of their tetrazine-TPEs by installing two tetrazines to two neighboring phenyl units rendering these probes two-point binders. One of their Tet-TPEs (TPE-Tz14) exhibited an enormous, 2400-fold fluorescence increase upon iEDDA and aggregation in the green range. Suitability of TPE-Tz14 in live cell no-wash labeling schemes was studied in mitochondria labeling experiments using a TPP-BCN derivative for organelle pretargeting (Table 1).

**Table 1:** Basic characteristics and reported application of selected fluorogenic tetrazine probes

Probe	_λabs_^a^ (nm)	_λem_^a^ (nm)	Proposed quenching mechanism	FE (I_IEDDA_/ I_Tet_)	Brightness of IEDDA product (εmax × Φ)a	Microscope application	Ref
Tet-V-SiR	645	667	PeT + tautomerism	22	6,530	GSDIM	[50]
Me-Tet-JF_646_	549	571	PeT	10	88,800	SIM	[74]
Me-Tet-ATTO655	663	680	PeT	13	37,500	confocal	[74]
Me-Tet-ATTO488	500	520	ET (FRET)	25	72,000	confocal	[74]
HD555	555	583	ET (Dexter)	123	57,100	confocal	[75]
HD654x	654	673	ET (Dexter)	33	n.d	confocal	[75]
HD653	652	674	ET (Dexter)	50	28,240	STED/SOFI	[75]
f-HM-SiR	653	669	PeT + tautomerism	10	48,100	dSTORM	[82]
Tet TO	526	548	IC + rotation	200^b^	50,000^b^	confocal	[83]
Dyad1	406 (552)	566	IC	16	n.a	STED	[76]
AzPaX-AA-MTet	560	590	ET	3	n.d	MINFLUX	[86]
TPE-Tz14	361	532	AIE + IC	2400	4,676	confocal	[93]

^a^of the IEDDA products

^b^in the presence of BCN and DNA

### Bioorthogonal Labeling Schemes Using Genetic Code Expansion Technology for Advanced Microscopy Applications

From the various bioorthogonal fluorescent probes available in the literature or even commercially, we can flexibly select the ones that suit best for our needs. Cell-impermeable dyes for labeling extracellular structures or cell-permeable ones to probe intracellular targets in vivo, or probes suitable for different kind of super-resolution imaging are all at our disposal. Herein we discuss recent advances in site-specific labeling of proteins for advanced imaging applications that apply minimal tags to endow the protein of interest (POI) with a complementary bioorthogonal function.

**Fig. 7:**
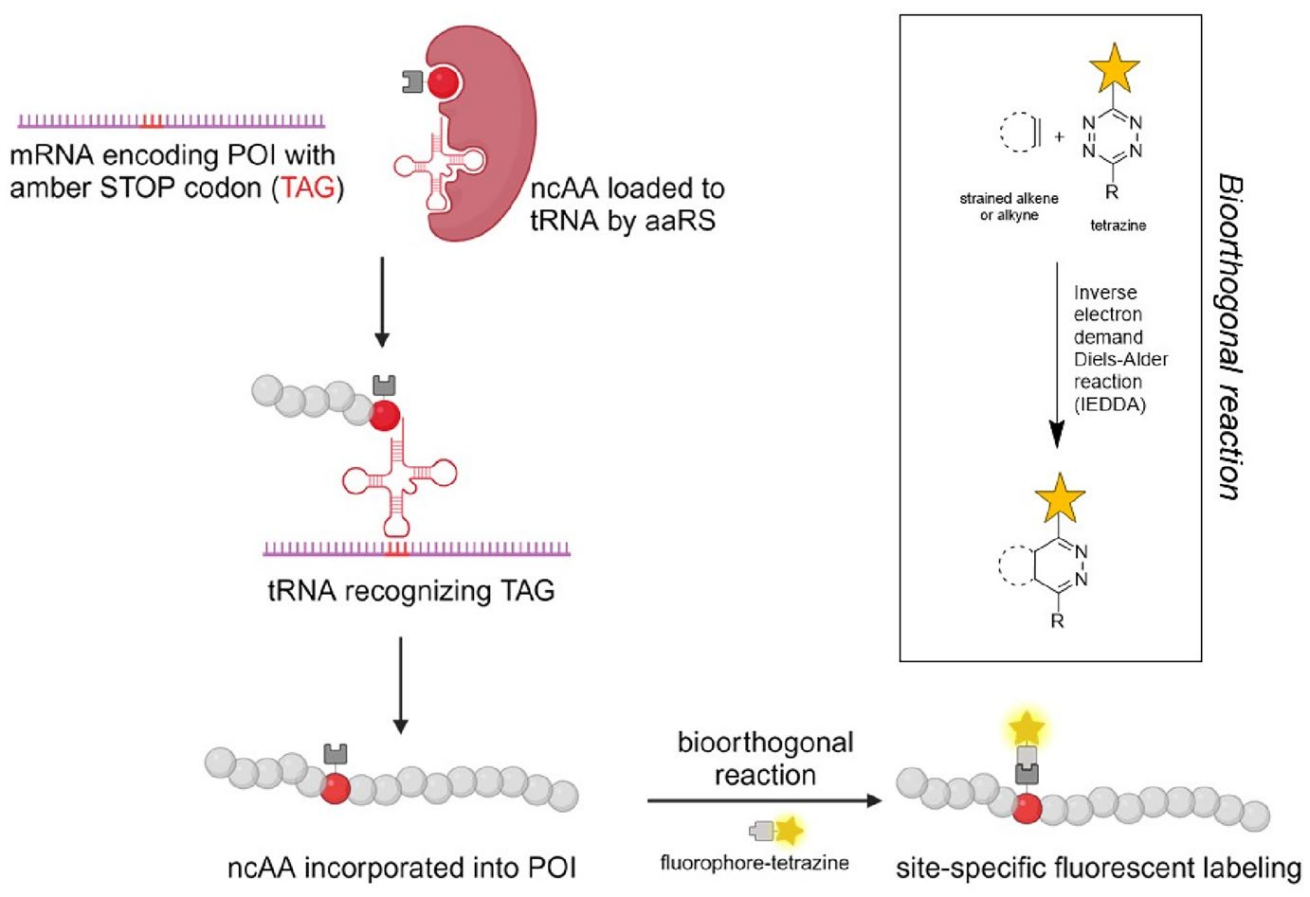
Genetic code expansion with non-canonical amino acids and subsequent fluorescent labeling for advanced imaging applications. A non-canonical amino acid (ncAA) bearing a bioorthogonal handle (strained alkene or alkyne) is supplied and charged onto an amber-supressor tRNA by an orthogonal aminoacyl-tRNA synthetase (aaRS). In the ribosome, the tRNA is decoded in response to an amber STOP codon (TAG) inserted in the mRNA encoding the protein of interest (POI). In response, the ncAA is built in the growing peptide chain and after protein translation, site-specifically labelled in a bioorthogonal inverse electron demand Diels–Alder (iEDDA) reaction with a fluorescent tetrazine derivative

Minimal tag bioorthogonalized handles can be introduced into proteins by genetic code expansion (GCE) technology [94–96]. In GCE, a non-canonical amino acid (ncAA) possessing a bioorthogonal side-chain is incorporated site-specifically into the sequence of a POI. By introducing a rare Amber STOP codon (TAG) to the gene of interest at the site of the proposed fluorescent modification, the incorporation of ncAA can be directed by a specific tRNA from *Methanosarcina* species that has been aminoacylated by an orthogonal tRNA synthetase (aaRS). Clickable ncAAs are recognized by a cognate engineered pyrrolysine tRNA synthetase (pylRS) with tailored binding pockets suitable to host eight-membered TCO-lysine or BCN-lysine derivatives [97, 98]. Subsequent iEDDA reaction between these genetically encoded clickable ncAAs and a fluorescent tetrazine derivative combines a fast, bioorthogonal reaction with site-specific labeling [99] (Fig. 7).

**Fig. 8:**
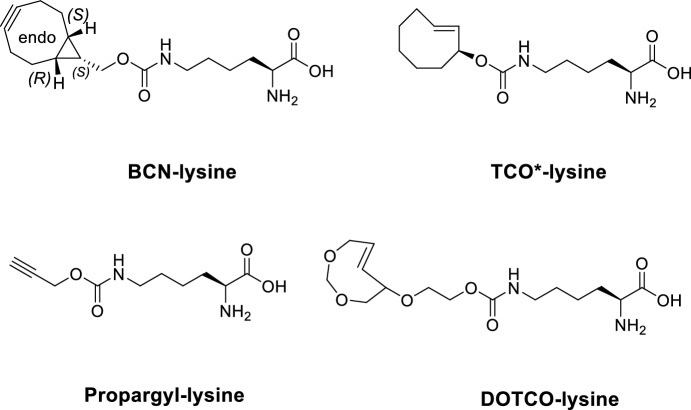
Genetically encodable non-canonical L-amino acids

Recently, several groups reported reengineered pylRS/tRNA pairs [100–102] ribosomes [103] and other synthetic organelles [104] to improve the efficiency of site-specific incorporation of ncAAs. Hell et al. combined a new, *N*-terminally optimized pylRS (chPylRS_2020_) with a previously engineered orthogonal tRNA to improve the incorporation efficiency of propargyl-lysine and BCN-lysine into β-actin in U2OS cell filopodia [101]. Subsequent labeling and MINFLUX imaging of the fluorescently tagged protein enabled a minimal distance difference between the true and the measured position of POI due to decreased linkage error. The minimal separation of fluorophores from the protein backbone allowed imaging of the 3D bundling architecture that was in excellent agreement with previously reported cryoelectron tomography data. Sauer et al. further pushed the resolution limit of GCE labeling by applying TCO*-Lys (TCO*A) tagging of subunits of multimeric proteins (GluK2 and GABAA receptors). Such precisely directed labeling of the proteins with Cy5-H-Tet allowed to take advantage on the strong dependence of the ON/OFF photoswitching kinetics on interfluorophore distance in the sub-10-nm range allowing to bypass the 10 nm resolution barrier [105]. These technological advancements significantly improved linkage-error free imaging based on GCE technology thus enabling researchers to answer some of the long-raised questions of cellular and neurobiology (Fig. 8).

Over the past decade, super-resolution microscopy revolutionized the way neuroscientists observe neuronal processes such as synaptic and axonal structure, protein arrangements in cellular subcompartments and patomechanism of neurological disorders [106–108]. However, given the nanoscale organization of synapses, one of the long-standing demands of neuroscience was the site-specific labelling of neuronal receptors enabling super-resolved imaging of synaptic activity. In their pioneering example Doose and co-workers managed to generate an amber mutant of the neuronal glutamate receptor, *N*-methyl-d-aspartate (NMDA) receptor subunit NR1 [109]. Although several antibodies are available for the NR1 and NR2 subunits of NMDAR, many of them target the intracellular regions thus not suitable for extracellular staining. Furthermore, bioorthogonal labeling of NR1^Y392TAG^EGFP mutant with TCO*-side chain labelled with Cy5-H-tet enabled studying receptor internalization in time-lapse imaging while antibody-stained NMDAR was completely immobile. Super-resolution imaging of the same mutant showed a homogenous membrane staining at significantly higher density compared to more spotted antibody-labeling.

**Fig. 9:**
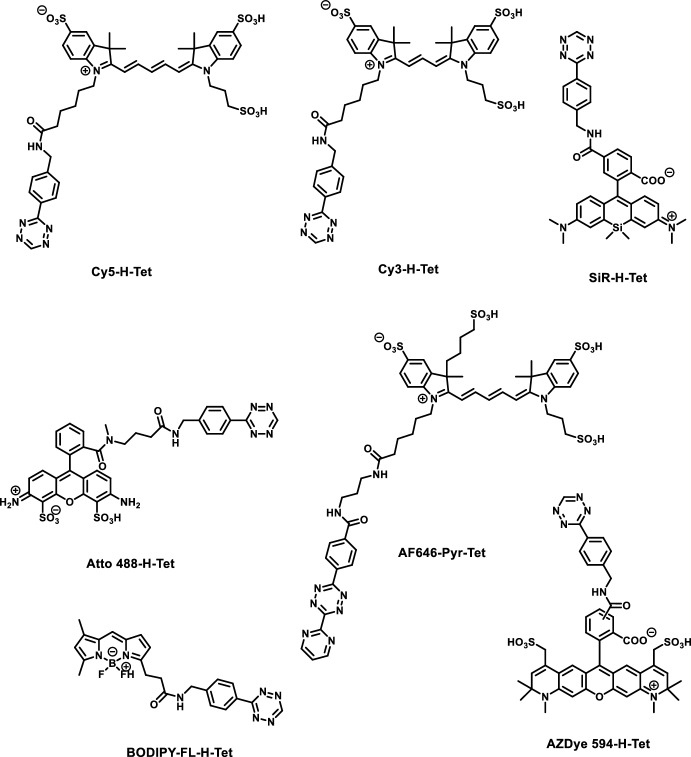
Fluorescent tetrazine derivatives

The high efficiency of GCE-mediated labeling in a crowded environment, such as in the case of extracellular protein domains in the synaptic cleft was further underlined by the groups of Werner and Sauer [110]. They generated multimeric, functionally intact membrane GABA-A receptor mutants with site-specifically incorporated TCO*-Lys. Cy5-H-Tet labeling allowed SIM imaging to localize GABA-A receptors on the neuronal surface revealing its accumulation at postsynaptic sites juxtaposing the presynaptic vesicular GABA transporter (vGAT) signal in primary cultured neurons (Fig. 9).

**Fig. 10:**
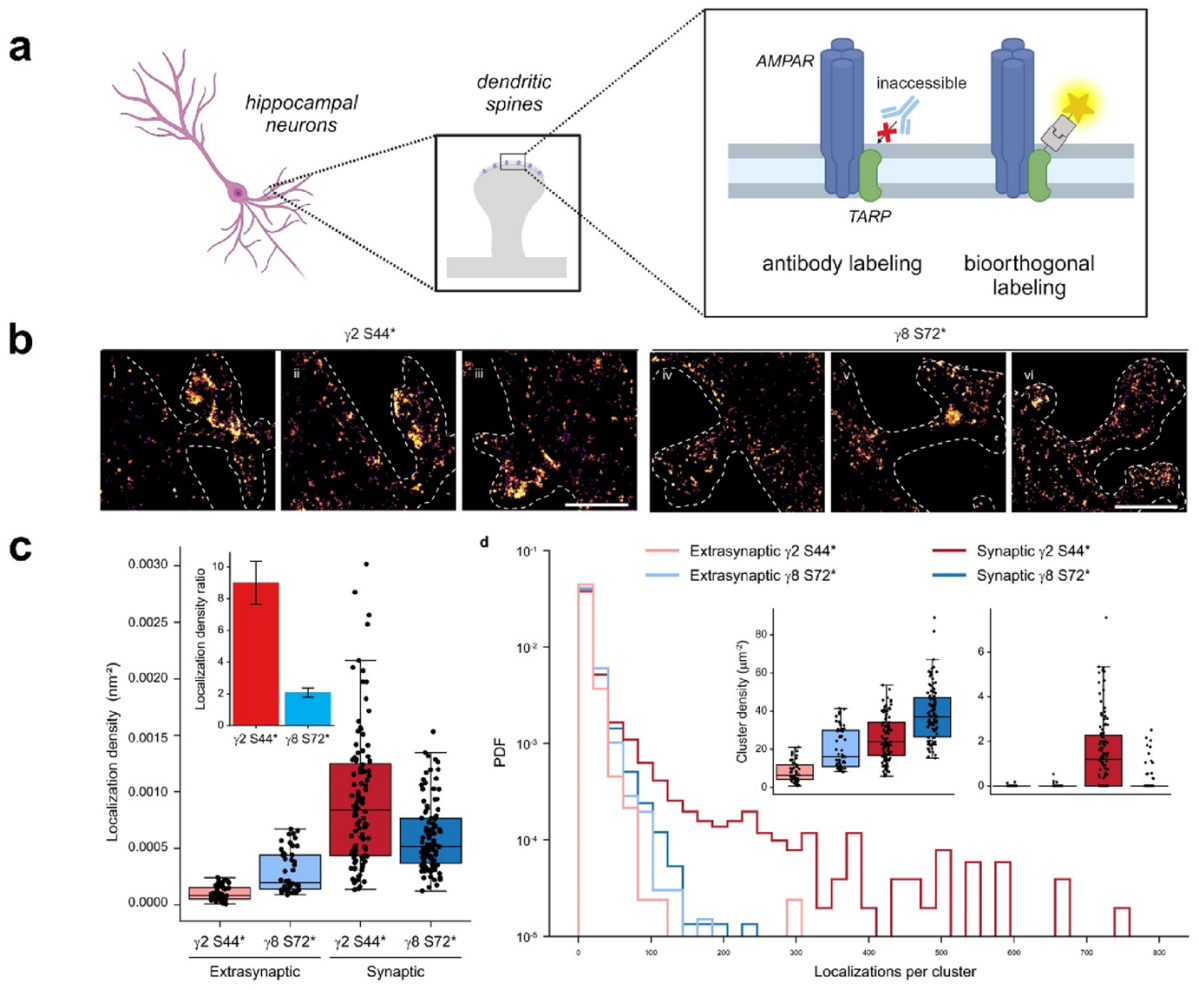
Superresolution imaging of nanoscale organization of neuronal proteins. **a** Choquet and Sauer presented fluorescent labeling of transmembrane AMPA receptor regulatory protein (TARP) which was impossible using antibodies due to epitope masking. This was circumvented with genetic encoding of TCO*-Lys into γ2 and γ8 TARPs and subsequent fluorescent labeling with AF647-Pyr-Tet. **b** dSTORM images revealed that AF647-Pyr-Tet clicked TCO*-modified γ2 accumulates in synaptic spines whereas γ8 shows a more even distribution between synaptic and extrasynaptic sites, **c** boxplots displaying higher synaptic localization densities for γ2 compared to γ8 and γ8 showed higher extrasynaptic localization densities in comparison to γ2. (Adapted from Ref. [111] with permission. Copyright © 2021 The Authors. Published by Springer Nature)

Pioneering demonstration of the real power of fluorescent GCE-aided labeling is from 2021 when the Sauer and Choquet labs performed bioorthogonal labeling of transmembrane AMPA receptor regulatory protein (TARP) [111]. TARP family members γ2 and γ8 are key regulators of AMPAR mediated synaptic transmission and plasticity. When attempting immunofluorescence-based labeling, the authors noticed that antibodies originally generated against extracellular domains of γ2 and γ8 failed to recognize their respective endogenous epitopes. They hypothesized that the close proximity of the extracellular domains of TARPs to the AMPA ligand binding domain (LBD) results in epitope masking thus rendering these sites sterically inaccessible to bulky antibodies. TCO*-Lys mediated fluorescent labeling on the other hand was possible allowing Cy3-H-Tet tagged TARPs to interact physically and functionally with AMPAR GluA1 subunit as revealed by FRET imaging and electrophysiology. The authors further investigated subcellular localization of TARPs with dSTORM microscopy to observe AF647-Pyr-Tet clicked TCO*-modified γ2 accumulation in synaptic spines whereas γ8 showed a more even distribution between synaptic and extrasynaptic sites (Fig. 10). This is in contrast to previous TEM findings, which could only detect these TARPs perisynaptically likely due to epitope masking, which was circumvented in GCE-based labeling.

The research group led by Nikić-Spiegel extended GCE-based click labeling to intracellular proteins of neurons in 2022 [112]. The authors genetically modified the neurofilament light chain (NFL) with TCO*-Lys at position 363 and conducted a thorough experiment to assess the source of background labeling in live cell intracellular applications, a well-known issue that limits the use of click labeling inside living cells [113]. They found a marked fluorescent background that originated from labeling non-incorporated ncAAs with SiR-H-Tet in the lysosomes. Also a diffuse background was revealed in the cytoplasm, which could partly be reduced by extended washing steps or by the use of somewhat more hydrophilic bioorthogonalized ncAAs, such as BCN-Lys or DOTCO [113]. Following careful optimization, the authors were able to click label two NFL populations in a pulse-chase manner in live primary cortical neurons using STED microscopy. They also applied two different tetrazine dyes (Atto488-H-Tet and SiR-H-Tet) to tell different populations of NFLs apart. More particularly, they could differentiate between NFLs synthetized previously during neuronal growth and transported distally along the axons and newly synthetized NFLs that were mainly present in the cell bodies. The same lab studied localization of axonal initial segment components (neurofascin and voltage-gated Na^+^ channel) in neurons using 3D dSTORM, a challenging task owing to the large size of the above-mentioned proteins (186 kDa and 260 kDa, respectively) [114]. The authors further increased the expression and click labeling efficiency using adeno-associated virus vectors together with TCO*-Lys incorporation and Atto488-H-tetrazine labeling.

**Fig. 11:**
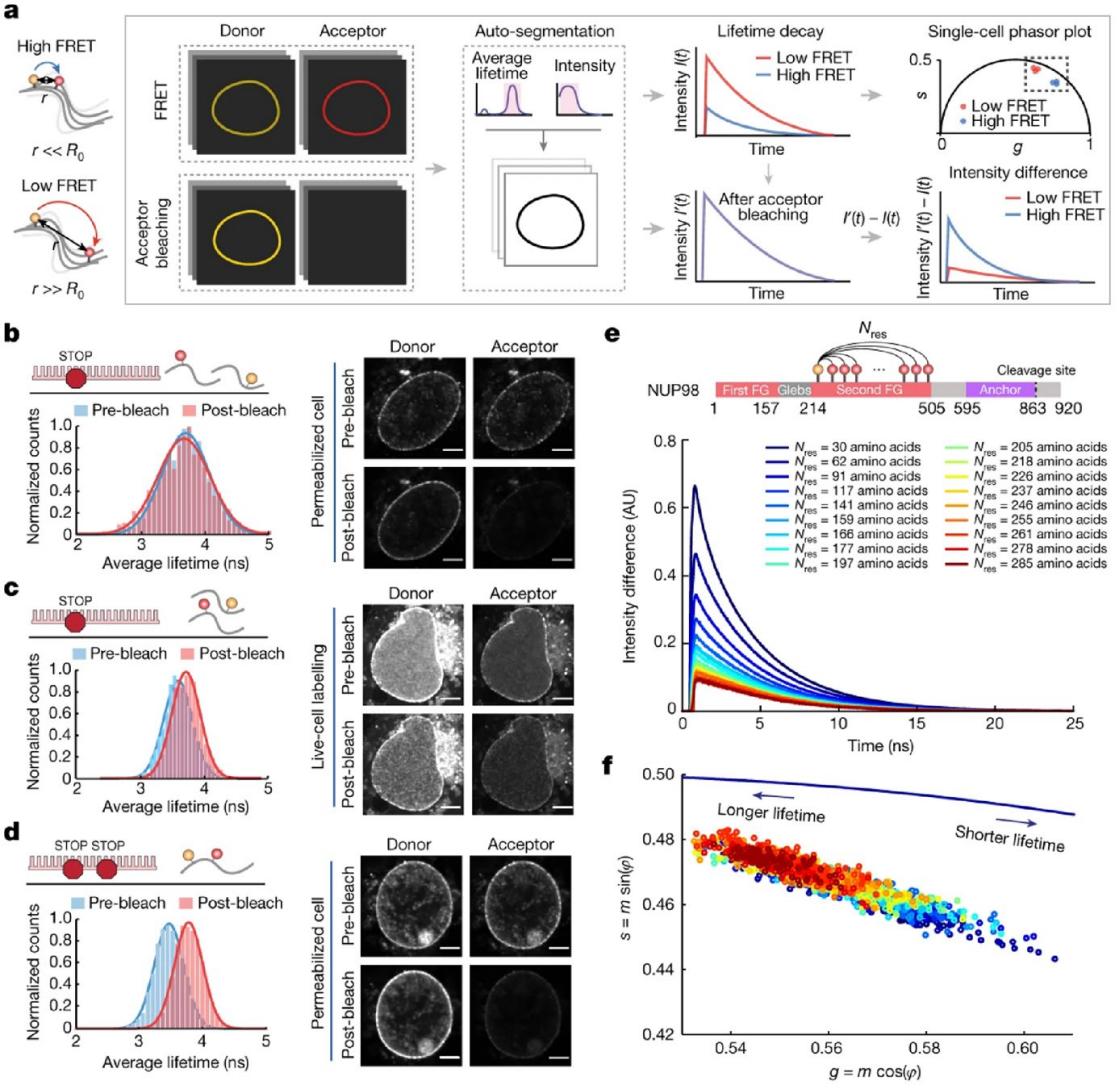
**a** Schematic of the FLIM–FRET analysis pipeline. Different chain segments of the NUP98 FG domain were labelled with a FRET dye pair, and the donor fluorescence intensity was measured on a cell-by-cell basis. Each nuclear rim was selected as a region of interest, and the measured donor fluorescence intensity profiles before and after acceptor photobleaching were extracted and analyzed. Acceptor photobleaching assays were performed for a single-amber-mutated sample (NUP98^A221TAG^) in permeabilized cells labelled with a AZDye594–LD655 mixture (**b**) and living cells labelled with a JF549–JF646 mixture (**c**). In b, the average fluorescence lifetime of the donor dye did not change before and after acceptor photobleaching, indicating the absence of intermolecular FRET. In c, the average fluorescence lifetime of the donor dye changed before and after acceptor photobleaching changed, indicating that intermolecular FRET was detected in highly overexpressing living cells with a bright nuclear rim. **d** Acceptor photobleaching assay was performed for a double-amber-mutated sample (NUP^98A221TAG–A312TAG^) labelled with the AZDye594–LD655 mixture. The average fluorescence lifetime of the donor dye changed before and after acceptor photobleaching, validating the presence of intramolecular FRET. For **b**–**d**, *n* = 5 experiments were repeated independently with the same conclusion; scale bars, 5 μm. **e** Fluorescence decay profile before photobleaching was subtracted from the one after photobleaching for the 18 different chain segments of the NUP98 FG domain. Each profile represents an averaged result of approximately 100 cells. The higher peak shows a greater difference in the intensity profiles, indicating higher FRET efficiency and smaller inter-residue distance. **f** Phasor plot showing donor lifetimes of the measured 18 chain segments on a single-cell basis (here approximately 2,000 cells in total), in which each point represents the fluorescence decay of one nuclear rim. The left-shifted points represent longer lifetimes. (Adapted from Ref. [116] with permission. Copyright © 2023 The Authors. Published by Springer Nature)

Visualizing previously “unseen” proteins is another appealing feature of genetically encoded click labeling schemes. Intrinsically disordered proteins (IDPs) are hardly compatible with protein fusion tags or antibodies due to their disordered structure that lacks a fixed tertiary structure and can adopt a range of various conformations [115]. In a very recent publication, the Lemke group mapped the molecular environment inside of mammalian nuclear pore complex (NPC) using synthetic biology and GCE-based bioorthogonal labeling methods [116]. The NPC forms a 120 MDa transport channel between the nucleus and the cytosol and is filled with hundreds of IDPs, called FG-nucleoporins (FG-NUPs). The authors studied FG-NUP98 and generated 12 amber mutants to optimize fluorescent labeling via TCO*-Lys. Tackling the challenging task of labeling intracellular IDPs brought about several technological advancements that was applied to e.g., film-like, synthetic, orthogonally translating organelles (OTOs) that form a protein translational machinery on the outer mitochondrial membrane and compensate for the background signal arising from the non mRNA-specific nature of conventional GCE technologies [104]. The authors incorporated TCO*-Lys at two sites of FG-NUP98 and attempted labeling with cell-permeable tetrazine dyes (e.g., JF646-H-Tet, SiR-H-Tet or TAMRA-H-Tet), which resulted in low signal-to-noise ratio. Therefore, they applied live-cell permeabilization with low dosages of digitonin and successfully labeled FG-NUP98 with two membrane impermeable dyes AZDye594-H-Tet and LD655-H-Tet that made up a FRET pair (a new photostable self-healing Cy5 conjugate [117] with low background (Fig. 11). FLIM-FRET measurements of the labeled proteins, allowed the authors to conclude that the channel provides a ‘good solvent’ environment that enables the FG domain to adopt extended conformations and thus control transport between the nucleus and the cytoplasm.

Thousands of microproteins (consisting of less than 100 amino acids) were recently described, however, their functional characterization is still incomplete due to their small size, which prohibits their tagging with fusion tags and to the fact that many of them are IDPs [118, 119]. Moreover, their physiological function needs to be assessed *in cellulo* to uncover complex formation with other proteins. These challenges call for the application of GCE-based labeling methods. The Slavoff group studied alt-RLP36, a previously not annotated 16-kDa protein translated from an alternative open reading frame (alt-ORF) of human ribosomal protein RLP36 [120]. To detect the subcellular localization of alt-RLP36, they introduced a BCN-Lys residue at position 18 and labeled it with SiR-H-Tet. Live cell imaging confirmed that alt-RPL36 is endogenously expressed and partially localizes in the endoplasmic reticulum.

In another example from 2019, the Hang lab studied an interferon-induced transmembrane protein (IFITM3), an important immune effector that prevents virus infections [121]. To address the IFITM3 function during virus entry, they site-specifically introduced TCO*-Lys to the POI and labeled it with BODIPY-FL-H-Tet. Such GCE-based fluorescent tagging and subsequent live cell imaging confirmed clear interaction of IFITM3-containing vesicles with viral particles and detected posttranslational modifications that regulate the function of the small protein. As these examples demonstrate, site-specific fluorescent bioorthogonal labeling schemes in combination with GCE can reveal previously unprecedented details of structure and function of microproteins and IDPs.

As presented in Refs 118 and 123, ncAA-mediated fluorescent labeling schemes facilitated the understanding of biological problems such as mechanism of nuclear transport via nucleoporins [116] and immune response of IFITM3 to viral particles [121]. There are further examples where bioorthogonal labeling schemes lead to structural and functional insights into proteins. Bioorthogonal fluorescent labeling was combined with biophysical single-cell methods to address common issues in receptor research, like diffusion, oligomerization states, and conformational changes [122–124]. Furthermore, click-tagged fluorescent receptors can also be applied to study receptor domain dynamics using bioluminescence resonance energy transfer (BRET) [125]. Using BCN-Lys mediated labeling by Weidemann et al. provided insights into interleukin-4 receptor alpha (IL-4Rα) structure and dynamics by screening receptor mutants bearing BCN-Lys in the extracellular domain [126]. Brightness-calibrated ratiometric imaging revealed site-specific variations of both labeling efficiency and IL-4 binding affinity and allowed detection of sub-angstrom shifts of receptor conformations in their native environment. Bioorthogonal labeling combined with superresolution imaging has advanced lipid research as well, revealing nanoscale lipid heterogeneity in living cells [127]. Furthermore, Lion et al. applied this approach to plant cells to reveal the zones of active lignification using Airyscan microscopy [128].

## Summary and Outlook

The above examples aim to provide the readers with an overview of recent developments in the field of bioorthogonally applicable probe developments and related imaging applications. Research directions in the last decade were mainly governed by the need for suitable probes to unleash the full potential of the emerged hardware developments. By now, some of the challenges that drove these fundamental research studies are certainly addressed. Yet, due to the ever developing area of superresolution imaging microscopy further challenges arose, which provoked the exploration of novel design strategies and smart solutions to develop non-invasive methods for the imaging of live cells or more complex structures to approach the resolution of electron microscopy. To increase the translational value of such bioorthogonal imaging techniques, effective modulation of probes in the NIR-I/NIR-II range is necessary [129]. To this end, design strategies not relying on quenching mechanisms need to be explored in the context of NIR emitting cores [130]. Alternative imaging modes may also be explored e.g., chemiluminescence [131]. Bioorthogonal conjugation armed chemiluminescence, however, should provide enough photons that can be captured within the short period of time provided. Capturing signals from such chemiluminogenic systems also requires certain technical advancements.

Although genetic code expansion assisted multicolor labeling schemes have been achieved [132, 133], it is still challenging to apply two different ncAAs as it requires two mutually orthogonal bioorthogonal reactions for labeling and two mutually orthogonal tRNA/RS pairs for ncAA incorporation at the same time [134, 135]. While mutually orthogonal ncAA-mediated click labeling remains challenging, GCE can be easily combined with other labeling strategies such as self-labeling enzymes or fluorescent proteins although at the cost of linkage error [136, 137]. Further to these, the existing mutually orthogonal bioorthogonal reactions possess substantially different reaction kinetics and laborious optimization process is required in terms of labeling concentration, time or administration order [138]. Also, with rare exceptions, these allow only dual color labeling of biological targets. Therefore, novel mutually orthogonal bioorthogonal reactions and bioorthogonalization strategies that address these limitations need to be explored.

As discussed, the use of minimal genetic tags was exploited e.g., in novel superresolution techniques to achieve a resolution of proteins that match the size resolution of cryoelectron tomography data. However, wider applications of site-specific bioorthogonal labeling of proteins is still hindered by technical limitations. For example, in genetic code expansion all the amber STOP codons will be transformed to a sense codon and as a result, proteins terminated by such a STOP codon will be affected upon incorporation of the exogenous amino acid and continue to express additional protein sequences. While it is expected that such C-terminally extended proteins degrade [139], they could potentially get labeled and contribute to the measured fluorescence signal. Moreover, it is challenging to remove unincorporated ncAAs from live cells. Since this can compromise the efficiency of the best-performing fluorogenic probe, development of more hydrophilic ncAAs is needed. Finding the proper position of the amber mutation of a given protein that retains its physiological structure and function while expressed in high yields is also a laborious process. N-terminal addition of a short amber STOP codon-containing peptide tag can be an alternative option as it was presented by the Elia lab [140]. Another issue to be resolved is the need for the overexpression of the proteins of interest as it may cause artefacts and cytotoxicity. Proof-of-principle studies to combine bioorthogonal labeling with genome editing tools such as CRISPR-Cas9 are promising directions as was presented [112], although recent advancements could possibly facilitate further refinement of the technology [141].

Further to these, implementation of the complementary bioorthogonal function that allow incorporation of minimal tag chemical reporters into target biomolecules other than proteins is still to be optimized. The success of genetic code expansion based incorporation of ncAA-based chemical reporters into proteins is yet to be achieved with carbohydrates, lipids and nucleic acids. In this respect the exploration of inherently accessible, selectively targetable bioorthogonal functions is promising especially toward the development of bioorthogonal labeling based diagnostic tools [142].
